# Reimagining Innovation in Health Equity: Making a Case for a Community embedded Participatory Learning Site for Adivasi Health Research

**DOI:** 10.36368/jcsh.v2i1.1102

**Published:** 2025-03-13

**Authors:** N S Prashanth, Sabu Kochupurackal Ulahannan, Anika Juneja, Tanya Seshadri, C Mahadeva, Muthaiah Venkategowda, C Made Gowda, Mathew Sunil George, Sumit Kane, Joris Michielsen, Sara Van Belle

**Affiliations:** 1 Health Equity Cluster, Institute of Public Health Bengaluru, Karnataka, India; 2 M. S. Swaminathan Research Foundation - Community Agrobiodiversity Centre, Puthoorvayal, Meppadi, Kerala; 3 Deva Soliga Cultural and Development Trust, Hanur, Chamarajanagar, India; 4 Jilla Budakattu Girijana Abhivruddhi Sangha, BR Hills, Chamarajanagar, India; 5 Ashoka Trust for Research in Ecology & Environment Field Station, BR hills, Chamarajanagar, India; 6 Health Research Institute, Faculty of Health, University of Canberra, Australia; 7 Nossal Institute of the Melbourne School of Population and Global Health, Australia; 8 Institute of Tropical Medicine, Antwerp, Belgium

**Keywords:** Adivasi, equity, participatory, realist, evaluation, community-based, interventions, India, Adivasi, equidad, participative, realista, evaluación, basado en la comunidad, intervenciones, India

## Abstract

**Introduction:**

This paper explores the development of the Realist Implementation Action Research Lab (RIAL), a participatory learning site aimed at addressing health disparities among Adivasi communities in India. Despite national health improvements, Adivasis face significant health inequities. RIAL employs a realist-inspired, theory-driven design to co-create solutions with communities, fostering collaboration among diverse stakeholders. The paper discusses insights from the establishment of RIAL, focusing on strategies implemented, opportunities identified, challenges encountered, and lessons learned to inform public health research and practice for Adivasi populations.

**Methods:**

We utilized processual analysis to examine the evolving dynamics of RIAL, combining historical and current documentation with collaborative team reflections. Context-Mechanism-Outcome (CMO) configurations were developed to tailor interventions to community needs and foster stakeholder engagement. These methods emphasized the contextual nature of health interventions, aligning with a systems-oriented, participatory approach. The establishment of RIAL involved consultations with community-based organizations, capacity-building workshops, and collaborative platforms to engage community leaders, healthcare providers, and policymakers, ensuring a participatory and contextually relevant foundation.

**Results:**

RIAL’s implementation highlighted the importance of reconfiguring power dynamics and fostering participatory processes. Strategies included co-design workshops, town hall assemblies, and capacity-building sessions, which enhanced community ownership and engagement. Challenges such as gender norms, logistical barriers, and resistance from non-Adivasi stakeholders were encountered, but iterative adaptation allowed for overcoming these barriers. Key findings included improved intervention receptivity, such as the successful relocation of a deaddiction clinic to a community-trusted NGO hospital and the implementation of sports-based psychosocial interventions for Adivasi youth, which demonstrated increased mental health awareness and reduced stigma.

**Conclusion:**

RIAL exemplifies the potential of participatory and context-aware methodologies in addressing health inequities among marginalized populations. Although scalability and resource availability pose limitations, this paper advocates for a shift from techno-centric solutions to those that are participatory, emphasizing sustained community engagement and co-creation of health interventions. The insights from RIAL’s implementation offer potential implications for adapting similar models in other contexts, aiming to reduce health disparities through inclusive research practices.

## Resumen

**Introducción:**

Este artículo explora el desarrollo del Laboratorio de Investigación-Acción para la Implementación Realista (RIAL), un espacio de aprendizaje participativo diseñado para abordar las inequidades en salud entre las comunidades Adivasi en la India. A pesar de los avances en salud a nivel nacional, los Adivasi continúan enfrentando desafíos significativos. RIAL emplea un diseño basado en teoría y en el enfoque realista para co-crear soluciones con las comunidades, promoviendo la colaboración entre diversos actores. Este artículo presenta estrategias implementadas, oportunidades identificadas, desafíos enfrentados y lecciones aprendidas para mejorar la investigación y la práctica en salud pública enfocada en los Adivasi.

**Métodos:**

Se realizó un análisis procesual para examinar la evolución de RIAL, combinando documentación histórica y actual con reflexiones colaborativas del equipo de investigación. Se utilizaron configuraciones de Contexto-Mecanismo-Resultado (CMR) para adaptar las intervenciones a las necesidades de la comunidad y fortalecer la participación de los actores clave. La creación de RIAL involucró consultas con organizaciones comunitarias, talleres de fortalecimiento de capacidades y plataformas colaborativas para asegurar una base participativa y contextualizada.

**Resultados:**

La implementación de RIAL destacó la importancia de reconfigurar dinámicas de poder y fomentar procesos participativos. Se aplicaron estrategias como talleres de co-diseño, asambleas comunitarias y sesiones de capacitación, lo que fortaleció la apropiación comunitaria. Se enfrentaron desafíos como normas de género, barreras logísticas y resistencia de actores externos, pero la adaptación iterativa permitió superarlos. Entre los principales logros se encuentran una mayor receptividad a las intervenciones, la reubicación exitosa de una clínica de rehabilitación en un hospital comunitario de confianza, y la implementación de intervenciones psicosociales basadas en el deporte para jóvenes Adivasi, que promovieron la conciencia sobre la salud mental y redujeron el estigma.

**Conclusión:**

RIAL ejemplifica el potencial de las metodologías participativas y contextualmente informadas para abordar inequidades en salud en poblaciones marginadas. Aunque la escalabilidad y la disponibilidad de recursos siguen siendo desafíos, este artículo aboga por un cambio de enfoques tecnocéntricos hacia soluciones co-creadas con la comunidad. Los aprendizajes de RIAL pueden guiar la adaptación de modelos similares en otros contextos, contribuyendo a la reducción de desigualdades en salud mediante prácticas de investigación inclusivas y sostenibles.

## Introduction

The Indian constitution recognises several communities as Scheduled Tribes (ST) for the purpose of affirmative action. Public health literature often identifies these communities as tribal communities or STs. However, many ST communities prefer self-identification as ‘*Adivasi*’, which asserts their identity as original inhabitant of the land [1]. In this paper we use *Adivasi*, as the preferred term to the Indigenous communities in our setting in southern India [2]. *Adivasi* communities constitute 8.6% of India’s population [3]. *Adivasi* people are heterogenous and with varying dynamics in power relationships with non-*Adivasi* communities across the country. In central Indian districts, despite forming the majority population, *Adivasi*s often find themselves politically underrepresented [4]. This contrasts starkly with the situation in the northeastern states, where the ST communities not only hold a numerical majority but also wield significant political influence. Conversely, in the southern states of India, the *Adivasi* community is both a demographic minority and politically less influential. This diverse representation and influence of the *Adivasi* community in different regions highlights the complex interplay of demographic and political factors within India’s social fabric [5–7]. Beyond political underrepresentation, these dynamics encompass economic marginalization, social exclusion, and restricted access to resources. *Adivasi*s frequently face land dispossession due to developmental projects or forest conservation policies, undermining their livelihoods and cultural identity. Social discrimination limits *Adivasi* access to education and healthcare, increasing their vulnerability to public health challenges and perpetuating cycles of poverty and poor health within India’s complex social framework [8–10].

Although health indicators have improved in many Indian states, *Adivasi* communities continue to face disproportionate health and development challenges, including higher rates of undernutrition, infectious diseases like malaria, leprosy, and tuberculosis, as well as significant income and literacy disparities. For instance, 39.2% of *Adivasi* households fall into the poorest wealth index category, and their literacy rate of 59% lags behind the national average of 74% [11–13].

The National Level Expert Committee on *Adivasi* Health has underscored that “one size fits all” health programs and policies are ineffective in addressing *Adivasi* communities’ complex and diverse health needs and healthcare barriers [11,13]. This observation aligns with the presence of 705 distinct ethnically diverse *Adivasi* groups in the country with unique social systems and cultural practices [3]. Therefore, the poor health and human development outcomes observed on a national scale among the *Adivasi* community are not evenly distributed across these diverse communities [14–16]. However, this diversity in the *Adivasi* context is not often acknowledged in the national health programmes and policies meant for them, such as Integrated Tribal Development Programs (ITDP) [17,18] and Janani Suraksha Yojana (JSY) [19]. These programs treat all *Adivasi* communities as being similarly “backward” and therefore tend to erase their distinctive characteristics [20,21]. *Adivasi* health interventions have also been criticised for lacking attention to diversity and sensitivity to cultural nuances, tokenism in the representation of community voices, and low priority for their unique health needs [22,23]. Additionally, most of the literature about *Adivasi* health inequalities has been largely cross-sectional prevalence studies as well as descriptive surveys that shed light on the gaps in access to health care [24]. Both state and central governments have paid minimal attention to community-based approaches and programs that address the social determinants of *Adivasi*-health or the determinants of health at the community level [11].

Therefore, devising and implementing flexible and adaptable community-based participatory health interventions is imperative. Using a ‘learning by doing’ approach that involves creating partnerships with community members, community organisations, service providers, and policy makers is an essential part of this approach [25,26]. An approach of this kind is crucial for addressing *Adivasi* communities’ diverse and complex health needs and ensuring that these changes are implemented in existing policy and practice, which is crucial for addressing inequities in *Adivasi* health [27]. This emphasis on participation and the pursuit of structural and social change through the empowerment of local stakeholders draws inspiration from the work of Paulo Freire and other scholar practitioners [25,26,28]. Community-based participatory approach recognises and addresses power imbalances and systemic inequalities. By actively engaging marginalised communities in the decision-making process, promoting their agency in research, and addressing historical injustices by transforming existing power dynamics, this approach aims to transform existing power dynamics and rectify historical injustices [29]. By engaging community members directly in decision-making processes, this approach strengthens their capabilities to make decisions. Therefore, interventions could be better tailored to meet the needs of *Adivasi* communities and are guided by their voices and directions. Additionally, it promotes collaboration between stakeholders, which is essential to the success and sustainability of any intervention. In addition, numerous studies have indicated that this engagement significantly contributes to the quality and sustainability of interventions [30–34].

The implementation of these approaches in Indigenous communities worldwide faces significant challenges and dilemmas, despite the existence of guiding principles and ethical frameworks [35]. These challenges involve coordinating research commissioned with academic focus, balancing the accessibility of research findings for community use while meeting academic standards, addressing inconsistencies in participatory approach applications, and navigating bureaucratic policies that may conflict with community priorities [29,32,36,37]. To advance the field, there is a need for ongoing training and mentoring, cultivating strong relationships with communities, respecting *Adivasi* knowledge, and continuously evaluating the research process with them [32,34]. In the Realist Implementation Action Research Lab (RIAL) framework, we prioritize a pluriversal approach, which acknowledges the coexistence of multiple valid knowledge systems and world-views within *Adivasi* communities. This approach involves not only adapting practical solutions that are contextually relevant but also deeply engaging with the *Adivasi* ways of knowing and seeing the world. For example, our participatory processes are designed to be inclusive of traditional knowledge, which influences both the problems we address and the solutions we co-create. By actively involving *Adivasi* community leaders and individuals in the research design and decision-making processes, we ensure that their worldviews are not merely included but are central to shaping the research agenda and outcomes.

### The RIAL Model

Building on the understanding of the complex challenges faced by *Adivasi* communities in India and the recognition of their diversity, the RIAL is being implemented with the Solega, an *Adivasi* community in the Chamarajanagar district of southern Karnataka, India. This community, characterized by unique cultural and social attributes, exemplifies the diverse nature of India’s *Adivasi* populations. The Solega community’s deep spiritual connection to forests, reliance on traditional ecological knowledge, and strong communal bonds significantly shape their engagement with interventions like RIAL, influencing both their adoption and adaptation [38]. The project’s focus on the Solega community allows for a tailored approach to address their specific challenges and needs. In RIAL, we integrate participatory approaches and realist inspired theory-driven design in co-producing interventions and plausible mechanisms and generates explanations for their apparent success or failures in different settings. This is grounded in a systems-oriented approach that problematizes systems and structures rather than focusing solely on individuals [39]. This systems-oriented approach aligns with complexity thinking, emphasising adaptive processes in dynamic contexts. Several core principles underpin this approach. First, it recognises the diversity among actors and involves a wide range of stakeholders as a strategy for achieving systemic change. Second, it places significant emphasis on triple-loop learning, which entails fostering emergent learning processes and creating positive feedback loops that address the inequitable distribution of power dynamics. The third loop holds the potential for emancipatory or transformative change, leading to sustained social transformation [40]. In RIAL, participatory approaches such as community-led decision-making, deliberative workshops, and co-design processes are employed to ensure inclusivity and representation of *Adivasi* voices. These methods are considered participatory as they actively involve community members in shaping interventions and ensure their contextual relevance [41]. It is a dynamic, iterative process that involves constantly adapting to feedback and contextual changes, reinforcing commitment to community-centric solutions.

The RIAL platform engages a diverse group of stakeholders, including Solega community leaders, members of community-based organizations (CBOs), policymakers, healthcare providers, and researchers. These stakeholders collaborate through structured processes such as periodic advisory meetings, deliberative workshops, and co-design sessions, ensuring that interventions are culturally relevant and community-driven. Core activities of the platform include prioritizing health challenges, gathering community feedback, and conducting capacity-building sessions to empower local leaders. These iterative processes are supported by continuous monitoring and action-reflection cycles, which adapt interventions to evolving community needs and ensure sustained engagement.

**Figure 1:**
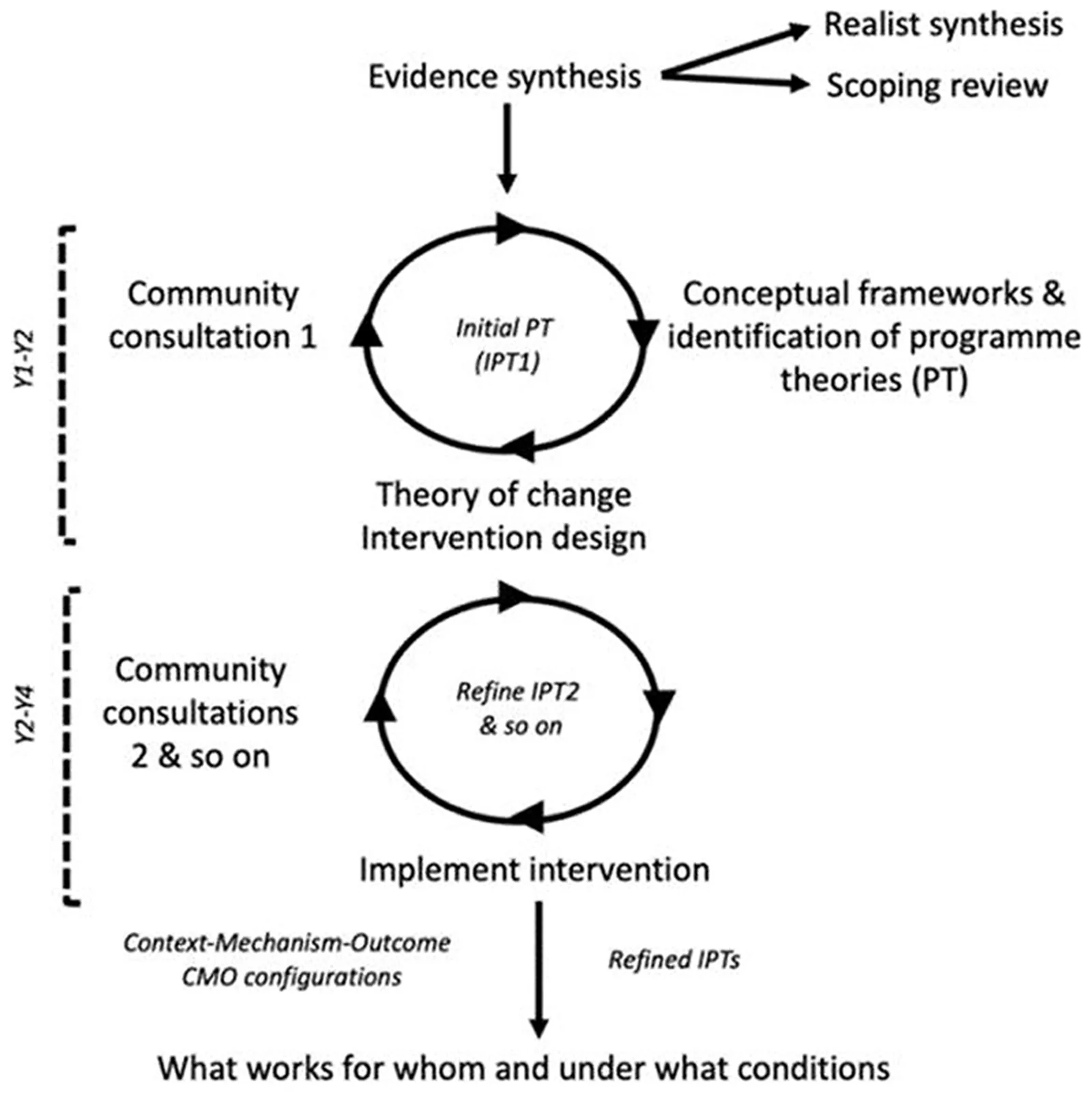
RIAL workflow illustration.

One of the major challenges of the participatory approach is that the outcome of such approaches depends on the interaction between the context and the intervention. As explained by Abildgaard et al., (2020) and Jagosh et al., (2015) [33,42], these interactions represent a dynamic process in which actors actively reason and adapt (or not) to the components of the interventions. This dynamism is central to understanding the variability in outcomes of participatory approaches. Building on this understanding, we have adopted a realist approach as this approach allows us to explore the nuances of these interactions, acknowledging that the success or failure of an intervention is intricately linked not only to its inherent qualities but also to the context in which it is implemented and is shaped by the actors and agents [43]. As Greenhalgh and Manzano explain, the realist approach recognises that, in addition to observable aspects such as space, place, and people, context encompasses the relational and dynamic elements that shape the mechanism through which an intervention operates. This approach also recognises the emergent nature of the context across different levels of the social system [44]. Hence, in the context of the cultural diversity and social stratification of the *Adivasi* community, a nuanced application of the realist approach is needed to improve health outcomes. In our intervention context, this means considering how the dynamics among *Adivasi* community members, healthcare providers, and policymakers at different levels shape program implementation and effectiveness. Acknowledging these complex relational mechanisms, which are integral to understanding and enhancing the impact of health interventions in diverse *Adivasi* settings, is a crucial adaptation in our approach to RIAL. By incorporating RIAL, we enhance our approach by tailoring interventions to the Solega *Adivasi* context. This involves conducting 2-3 action-reflection cycles with support groups and utilising qualitative methods such as in-depth interviews to refine interventions (Figure 1).

Figure 1 illustrates the iterative process of developing, implementing, and refining interventions in RIAL. The process begins with evidence synthesis through realist and scoping reviews, which inform the development of an initial program theory based on CMO configurations. This realist evaluation approach ensures that interventions are empirically validated and contextually suitable, addressing the dynamic interplay between context and intervention in *Adivasi* communities. Community consultations guide the refinement of conceptual frameworks, theory of change, and intervention designs. These designs are tested and iteratively refined through additional cycles of consultation and implementation, ultimately identifying “what works, for whom, and under what conditions.

### The initial programme theory

“If researchers establish trusting relationships with *Adivasi* community-based organisations (CBOs) and individuals through small-scale actionable research projects, then it is anticipated that a collaborative platform will be created. This platform could enable *Adivasi* CBOs, leaders, and individuals to claim space and feel empowered to voice their opinions on healthcare programs and services. Consequently, this process is expected to significantly enhance the nature of participation and engagement of the *Adivasi* people in healthcare programs” [45]. From the realist perspective, the context in this framework includes the specific socio-historical circumstances of the *Adivasi* community, particularly their experiences with affirmative action programs and the forest conservation regime. These experiences have shaped their engagement in various sectors, including healthcare. Understanding and acknowledging this context is vital for the successfully implementing the RIAL program. The mechanism in this framework is twofold: resources and reasoning [44]. The resources refer to RIAL’s collaborative platforms, designed to facilitate engagement and dialogue among stakeholders. Participants perceive a “collaborative platform” as a shared space for equitable dialogue, joint decision-making, and mutual accountability. Criteria such as inclusivity of diverse voices, regularity of stakeholder interactions, and the empowerment of *Adivasi* leaders to influence program decisions qualify these platforms as collaborative [46]. The reasoning aspect relates to fostering a sense of inclusion in decision-making, which is anticipated to trigger active community participation in program design, implementation, and evaluation. The expected outcome of this approach is the establishment of a platform that is actively claimed and utilised by *Adivasi* CBOs, patients, and citizens. This participatory space creation occurs within the aforementioned socio-historical context, acknowledging that *Adivasi*’s participation in various programs has been adversely conditioned by their historical engagement in non-health programs such as forest conservation regimes [47]. While this indeed seems to be intuitive, it remains an important hypothesis to study and test. This approach is especially relevant considering multiple settings where empowering and free healthcare programs and services are apparently not often engaged with by communities, as noted by George et al. (2020) [22]. Therefore, initial programme theory emphasises the importance of creating effective participatory spaces grounded in trust and mutual understanding to ensure meaningful engagement of the *Adivasi* community in healthcare programmes. Despite the widespread application of participatory processes by civil society, systematic descriptions of the methodological approaches underlying their practice in public health are scarce [48]. Hence, in this paper, we describe an ongoing process to co-create a participatory platform that is implementing the RIAL intervention. We also discuss the challenges, opportunities, and shortcomings in order to inform other participatory implementation research endeavours in *Adivasi* health or related areas.

## Methods

### Study Design and Context

In this study, we used processual analysis as the primary method to understand the evolving dynamics of RIAL in *Adivasi* communities. SKU, PNS, TS, CM, MV, and AJ, who are co-authors of this paper, also served as key stakeholders in the implementation process, contributing direct insights from their dual roles as researchers and active participants. The study was conducted in the Solega *Adivasi* community in the Chamarajanagar district of Karnataka, India. The implementation of RIAL was built on a foundation of enduring relationships and mutual trust between the leaders of the Zilla Budakattu Girijana Abhivrudhhi Sangha (Sangha), a district-level organization advocating for the Solega community, and Vivekananda Girijana Kalyana Kendra (VGKK), an NGO dedicated to the advancement of the Solega people since 1981 [49]. The Solega community’s federated organizational structure spanning village, sub-district (taluka), and district levels was a critical factor in shaping the participatory methods of RIAL [50].

### Processual Analysis

Processual analysis was conducted through iterative cycles of team reflections, detailed documentation of intervention activities, and thematic analysis of collected data to identify patterns of change and adaptation over time [51]. The analysis focused on the period from the inception of our field learning site in 2014 through its subsequent evolution into RIAL up to 2022. Five relevant peer-reviewed papers, co-authored by members of the implementation team (TS and PNS), were reviewed and identified as critical for understanding the project’s evolution.

### Collaborative Reflections and Framework Development

Collaborative reflections were conducted through iterative discussions involving the key implementation team. These discussions were held in both English and Kannada to facilitate inclusive participation of scholars and community representatives. Translations between the two languages ensured clarity and accessibility for all participants, and detailed notes were systematically recorded to maintain accuracy and comprehensiveness. Insights from these reflections contributed to the development of the RIAL theoretical framework. The framework was refined through an iterative process of dialogue and consensus-building among scholars and community stakeholders, integrating contextual analysis and stakeholder feedback. Realism was applied by examining the interactions between context, mechanisms, and outcomes to understand how and why interventions succeed or fail in different contexts, ensuring the framework’s practical relevance and adaptability.

### Development of the RIAL Learning Site

**Figure 2:**
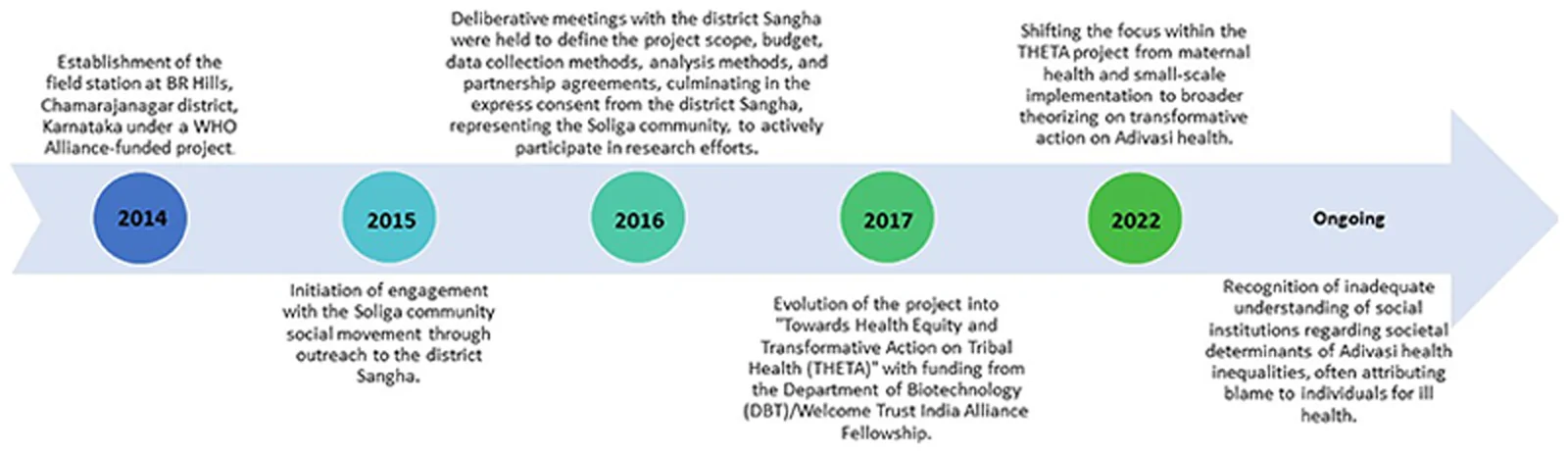
Key Phases in the Development of Adivasi Health Initiatives.

The development of RIAL began as a time-bound participatory action research project addressing the social determinants of maternal and child health inequities in the Solega *Adivasi* community. By leveraging the relationships and organizational structures described earlier, the RIAL evolved into a sustainable learning site [52]. This evolution reflects a gradual shift from addressing specific health needs to theorizing transformative action on *Adivasi* health. Figure 2 summarises the timeline and key phases of this evolution and highlights the iterative development of the learning site. RIAL employed participatory approaches such as community-led decision-making, deliberative workshops, and co-design processes. These methods ensured inclusive representation, enabling *Adivasi* community members to actively shape interventions and decisions. This approach aligns with established principles of participatory research, which emphasize collaboration and inclusivity to address systemic inequalities effectively [31].

### Ethics approval and consent to participate

Ethics approval for the interventions conducted in this study was granted by the Institutional Ethics Committee (IEC) of the Institute of Public Health, Bengaluru (IPH/22=23/E/324).

## Results

The implementation of the Realist Implementation Action Research Lab (RIAL) represents a dynamic and evolving effort to address health inequities among the Solega *Adivasi* community. Grounded in participatory approaches and guided by a realist framework, RIAL builds on pre-existing relationships and community structures while adapting to challenges through iterative reflection and collaboration. This section presents the outcomes of RIAL’s implementation, highlighting its impact on fostering transformative progress in health equity, addressing systemic barriers, and developing contextually relevant health interventions. The findings also illustrate how contextual factors, such as gender norms, socio-economic disparities, and historical discrimination, have influenced the mechanisms and outcomes of RIAL, shaping its ongoing evolution.

### Revision of the community-based approach – setting up RIAL

Our reflection on participatory engagements with the Solega communities and Solega community social movements has prompted us to revise our engagement with the *Adivasi* communities. We realised that working with communities to catalyse community-level mobilisation, defined as collective actions led by community members to address shared challenges, is critical for achieving transformative progress in *Adivasi* health equity. This mobilization was facilitated through participatory methods such as deliberative workshops, focus group discussions, and collaborative decision-making processes that actively involved diverse community representatives, including women and socio-economically disadvantaged individuals. These participatory approaches ensured that interventions were tailored to the unique sociocultural and economic context of the Solega community. They also emphasized inclusivity, allowing marginalized voices within the community to actively shape the interventions and decision-making processes.

However, the challenge of ensuring inclusive representation of women and socio-economically disadvantaged individuals within the community persists in our work too. Barriers such as community gender norms and geographical remoteness significantly impacted the diversity and extent of community participation in the collaboratively designed interventions (53). For instance, gendered expectations that women prioritize household chores like cooking, cleaning, and childcare over leisure activities often limited their availability for participation. Additionally, cultural perceptions regarding women’s safety and mobility further restricted their involvement, as families were hesitant to allow women and girls to travel or spend time outside the home. It is also worth noting that some of these barriers are influenced by the gendered roles of non-*Adivasi* society, particularly in instances where *Adivasi* women leaders are expected to come to cities or travel long distances. In such contexts, the prevailing gender norms of non-*Adivasi* people in cities and towns tend to shape how gender is practiced among *Adivasi* leaders. On the other hand, within smaller gatherings in *Adivasi* settings, these external influences are less significant, and the practice of gender roles within *Adivasi* society remains distinct and less impacted by these outside norms. Reflecting on our previous assumptions, learning from these shortcomings, and incorporating community voices, we have revised our approach, reconceptualised our strategies, and developed plans to foster the inclusion of diverse participants from the *Adivasi* communities with whom we collaborate. Community voices, which were critical in shaping RIAL, were obtained through iterative consultations with community leaders, members of the Sangha, and local stakeholders during regular meetings and workshops. These inputs included prioritization of health challenges, feedback on intervention design, and culturally appropriate solutions to address specific health issues. For example, during a deliberative workshop held in 2016, community members analyzed survey data and prioritized themes such as addiction recovery and mental health interventions, providing valuable direction for subsequent phases of RIAL. This shift in approach has led us to conceptualise RIAL as a platform in its current form.

### Reimagining power relationships

**Figure 3:**
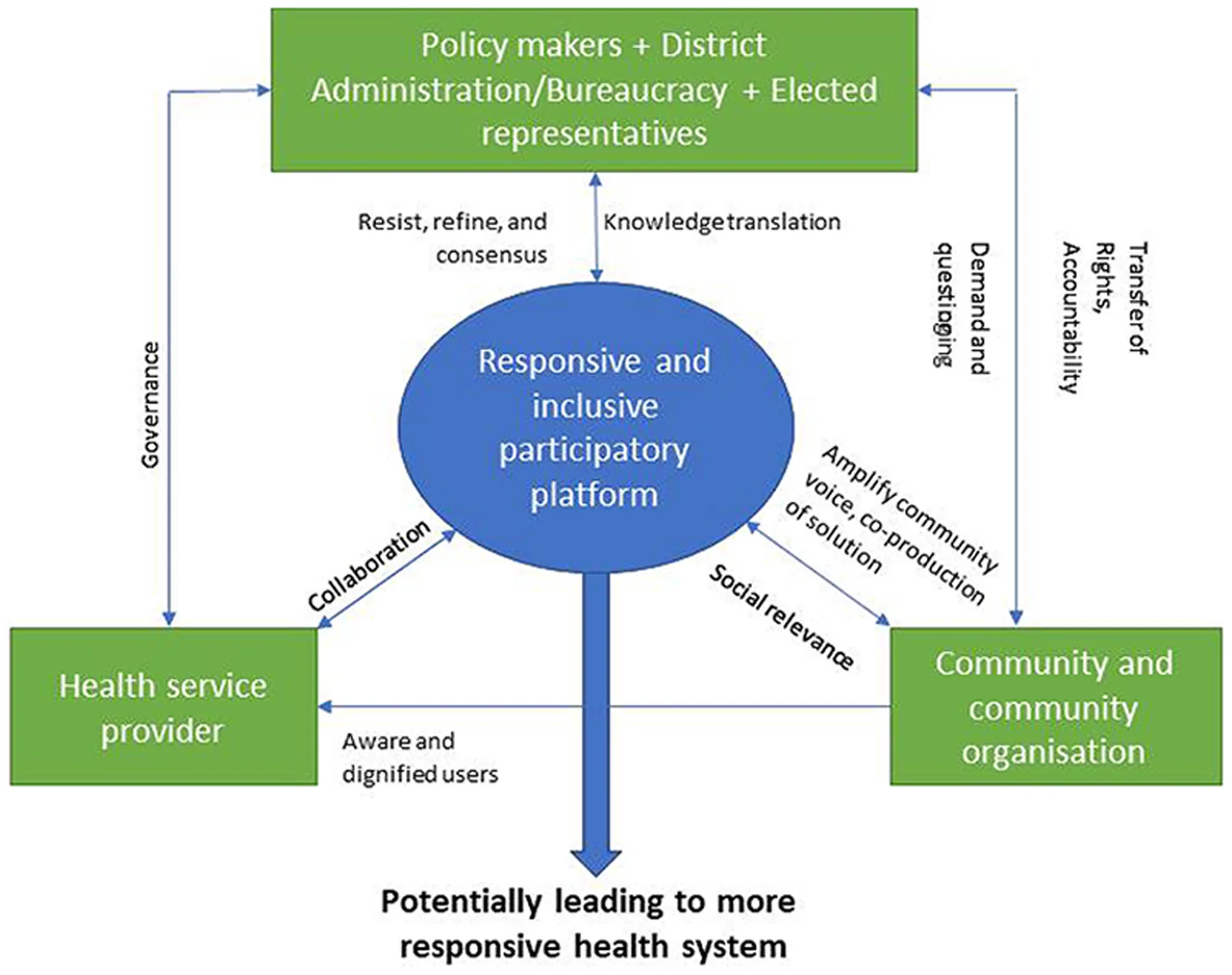
Proposed reimagination of the relationship dynamics between potential key actors in RIAL.

There is a growing acknowledgement in the global health literature, as indicated by Pai et al. (2018), that technological innovations not addressing social determinants of health (SDH) and their inequalities into account often fail to improve health outcomes at the population level (54). In this context, innovation in *Adivasi* health is not primarily about introducing new health technology but rather is perceived as a process, a novel approach to engagement. This involves reimagining and rebuilding relationships between the *Adivasi* community, community-based organisations, service providers, and policymakers. The strategy is multifaceted, emphasizing collaboration, amplification of community voices and constructive engagement with resistance (Figure 3). This figure illustrates the proposed RIAL model as a responsive and inclusive participatory platform designed to foster collaboration between key stakeholders, including policymakers, district administration, elected representatives, health service providers, and community organizations. The platform facilitates knowledge translation, governance, and accountability by amplifying community voices, co-producing solutions, and encouraging demand and questioning from the community. By integrating social relevance, collaboration, and mutual accountability, the model aims to establish a health system that is not only more responsive but also more inclusive, ensuring that the needs and aspirations of the *Adivasi* communities are central to decision-making processes. The flow of interactions depicted in the figure highlights how these elements come together to drive transformative change in the health system. This approach, aligning with Mouffe’s (1999) agonistics philosophy, underscores the importance of consensus-seeking and conflict-engaging deliberations for integrating diverse perspectives into health care decision-making. This is particularly relevant in RIAL, where we emphasise methods that allow for the reconfiguration of local power relationships, which are deemed essential for the success of participatory health interventions with *Adivasi* communities.

Reconfiguring power relations is vital, as highlighted by Friedman and Gostin (2017), due to the impact of power imbalances in healthcare that lead to inequitable access and a deficit of trust between healthcare providers and *Adivasi* communities [55]. In RIAL, this was achieved through cocreating platforms such as advisory boards and committees where decision-making authority was consciously ceded to *Adivasi* leaders, and initiatives like the *Adivasi* Arogya Samvada, an annual town hall-style assembly, facilitated active dialogue and prioritized *Adivasi* leadership in meeting agendas. The implementation of these methods involved iterative consultations, capacity-building workshops, and agreements with stakeholders, but challenges such as resistance from non-*Adivasi* stakeholders and logistical constraints were encountered. Despite these challenges, the integration of *Adivasi* individuals into diverse roles within the project fostered inclusivity and strengthened trust, making their perspectives central to the research process. This reconfiguration aims to create a community-centred health system in which *Adivasi* communities are participants and active decision makers in their health care, ensuring that their voices are effectively heard and contribute to fostering social change by reducing inequities. This participatory process is about reimaging space and voice, requiring a shift in power dynamics. In RIAL, this is achieved by cocreating platforms where *Adivasi* leaders play influential roles, such as on advisory boards or committees where influence is consciously ceded in favour of their inclusion. Initiatives such as the ‘*Adivasi* Arogya Samvada,’ an annual town hall-style assembly, facilitate this process by fostering active dialogue and disagreement (reimagination of voice) and prioritising *Adivasi* leadership in meeting locations and agendas. We also employed as many *Adivasi* individuals as possible within the project. Including *Adivasi* individuals in diverse roles within the project is a prime example of reimagining the space for participatory research. This approach facilitates the seamless integration of experienced community-based organisations (CBOs) members into the research team. It also repositions researchers into the role of learners in the context of *Adivasi* health research, thereby fostering a more inclusive and collaborative environment. This reimagined space acknowledges and actively leverages the expertise and insights of the *Adivasi* community, ensuring that their perspectives are central to the research process [56,57]. Our engagement with diverse stakeholders, including community members, policymakers, administrators, and healthcare providers, revolves around amplifying *Adivasi* voices and cocreating solutions. This ensures that their needs and aspirations are central in decision-making processes. Following Mouffe’s concept of agonistic pluralism [58], our approach incorporates both consensus-building and conflict-engaging deliberations. Mouffe advocates for the harnessing of political disagreement as a means to enhance participatory engagement. By doing so, we aim to foster a balanced and inclusive relationship with all levels of governance, acknowledging that disagreement is not a hindrance but a catalyst for deepening democratic processes and improving health interventions.

### Practising the learning site: the case of addiction

The results from the THETA project indicated that the incidence of tobacco and alcohol use in various *Adivasi* communities across India are twice as high as that in nearby non-*Adivasi* communities [59]. This could lead to lasting, multigenerational impacts, exacerbating existing inequalities [60]. Moreover, support services for addiction recovery and quitting substance use have yet to be implemented in rural areas [61]. Hence, in RIAL, we identify evidence-informed group interventions for peer support for harmful substance use and implement them using participatory methods. The *Hosa Jeevana Tobacco cessation clinic* was initiated to establish a tobacco cessation clinic in a community-based manner anchored at Taluka Hospital (subdistrict) with linkages to primary health centres. A Memorandum of Understanding (MoU) was signed with Taluka Hospital, and the clinic functioned half a day every Tuesday, from morning to midday until December 2022. Despite our best efforts, we could not gain substantial acceptance of this clinic from the patients or the necessary support from the government in posting counsellors to the clinic. Based on internal reflection on this with officials and community representatives, we have moved the clinic and the community-based component to the VGKK NGO hospital where the clinic runs weekly on Fridays for half a day and has already begun enrolling people. This NGO hospital is deeply rooted in the community due to its focus on *Adivasi* community development and the integration of traditional and modern medical practices [50]. Their approach is more aligned with the specific needs of the *Adivasi* community, which makes them an ideal setting for the implementation of the *Hosa Jeevana Tobacco cessation clinic*.

Evidence on de-addiction interventions among similarly marginalised communities in other parts of the country shows that de-addiction interventions can be successfully delivered by lay counsellors, who are typically community members with some level of training but without formal psychological or medical education [61]. These findings hold significant relevance for *Adivasi* communities in our project area, where access to professional health care is limited by distance and cost. Training local community members as lay counsellors may lower these barriers, potentially enabling the program to be making the program more accessible and culturally sensitive. Second, the community-based nature of our intervention has the obvious advantage of being logical concerning the socio-historical and cultural context. It enables the programme to be set in the context of existing norms, customs and values, which could be invaluable for the intervention’s acceptability, relevance and ‘buy-in’. Thus, drawing from this evidence, a community-based alcohol and tobacco de-addiction program is being co-designed. We expect that this approach will increase accessibility, reduce costs, and allow for cultural sensitivity and tailoring, which are crucial for the successful delivery and uptake of such a program in these communities. Incorporating the RIAL approach into this intervention, we initiated participatory design workshops involving health workers and service users. This commenced with embedding a research fellow with a psychiatric social worker at the RIAL clinic. Building on the RIAL’s overarching initial programme theory, our next step is to develop a specific initial programme theory for addiction clinics within the *Adivasi* context. This involves a process of trying, refining, and learning how to effectively run an addiction clinic tailored to the unique needs and circumstances of the *Adivasi* community. The ultimate goal of this intervention is to establish a plausible implementation model for community-based de-addiction programs in *Adivasi* settings.

While hospital- and community-based de-addiction programmes focus on current users, preventive and wellness-oriented approaches using sports-based mental health interventions constitute the second component of our programme. Our review of the literature on *Adivasi* health inequities suggests that the stigma and discrimination that *Adivasi* communities face in their everyday life [62,63] can cause a negative perception of self, resentment, loneliness, anger, shame, and anxiety, leading to psychosocial disability in their everyday life [64,65]. These adverse life conditions of the *Adivasi* population are the leading cause of the harmful use of alcohol and tobacco among the people affected [65,66]. The combination of poor mental health, substance abuse, and other socio-economic disadvantages creates a vicious cycle, aggravating the existing health inequalities [16,67,68]. The findings further recorded the initiation of alcohol and tobacco use at very early adolescence (teenage years) among *Adivasi* youth (59). Hence, we implemented a sports and life skills-based psychosocial intervention called ‘*OneAll*’ to prevent alcohol and substance use and promote positive mental health among the *Adivasi* youth in Chamarajanagar district in collaboration with the One All Trust, a community-based organisation that has been implementing this intervention among the *Adivasi* youth in the Gudalur Taluk of the Nilgiris District of Tamilnadu (One All Trust https://one-all.in/about/philosophy/). Early adulthood is critical for developing protective psychosocial characteristics to break the cycle of poverty, social exclusion and mental ill health (69). Through this initiative, we aim to mitigate the adverse effects of stigma, discrimination, and socio-economic challenges faced by the *Adivasi* community and inspire wellness-oriented youth role models in the community who could help reclaim the diminishing solidarity mechanisms among the *Adivasi* community and dissuade the community from alcohol and substance use.

## Discussion

In this paper, we describe our journey to establish a new RIAL in the Chamarajanagar district of Karnataka. RIAL employs a realist methodology that acknowledges the varying success of interventions across contexts, combined with participatory approaches that attempt to rebalance power dynamics and increase inclusion. The interventions of this programme specifically aim to address high rates of substance use among the *Adivasi* community, improve overall health outcomes, and ultimately reduce systemic health inequities. Additionally, there is a keen focus on addressing psychosocial disability and preventing substance abuse among *Adivasi* youth. These features highlight the importance of understanding the unique needs, circumstances, and cultural nuances of the *Adivasi* communities and represent a significant strength of the approach described in this paper.

This study provides evidence to validate the initial program theory of RIAL, which hypothesized that creating participatory spaces, reconfiguring power relationships, and fostering local leadership would lead to more inclusive and responsive health systems. For example, the *Adivasi* Arogya Samvada and the integration of *Adivasi* leaders into advisory boards illustrate how participatory approaches (Mechanism) in a supportive cultural and organizational context (Context) led to increased representation, community empowerment, and improved trust and engagement (Outcome). Similarly, the adaptation of the tobacco cessation clinic highlighted how tailoring interventions to cultural and logistical realities facilitated better community participation and ownership. This aligns with findings from our earlier work using realist interviewing, which emphasized its value in refining program theories and ensuring community voices are integral to intervention design and implementation [70].

Despite these significant strengths, there are some potential limitations to our study. One of the challenges is that extent of direct scalability of these interventions due to each community’s diverse and unique nature. However, contextually relevant interventions such as RIAL by definition may not be universally applicable and require context-specific approaches and resources across different regions or larger populations [71]. Furthermore, the intricate interplay between multiple stakeholders and the need for additional training for local community members and healthcare providers in new regions present additional hurdles [72]. However, these limitations do not negate the value of contextually relevant interventions such as RIAL. Instead, they highlight the need for careful and deliberate adaptation when scaling these interventions, maintaining their relevance while modifying them for different communities and settings. The potential for adaptation of RIAL model in diverse contexts rests on a nuanced understanding of local realities and leveraging universal principles of participatory engagement. To this end, the RIAL insights are not prescriptive but rather serve as a flexible guide, underpinned by a realist evaluation that asks what works, for whom, and in what circumstances. This approach necessitates a dynamic and reflective process of learning and adaptation, with the transferability of insights contingent upon the engagement of local stakeholders in tailoring interventions. Having a model that allows contextualised solutions, flexibility, and decentralised implementation and decision-making is crucial to achieving equitable health for *Adivasi* communities [73]. This approach helps counter the growing trend of prioritising scalability as a rationale for adopting decontextualised solutions, especially in communities experiencing social exclusion [74]. In our view, using scale only as a metric for success undermines the ability to address deeply rooted historical processes and injustices that have shaped these communities. While there are acknowledged limitations related to scalability, the demonstrated potential for positive impact through culturally sensitive, community-based participatory approaches underscores its value. An overemphasis on scalability often fails to capture the complexity and specificity needed to enact meaningful, long-lasting change.

### Conclusion

In conclusion, this paper delineates the effectiveness of RIAL in fostering health equity among the *Adivasi* communities by emphasizing participatory and context-aware methodologies. The insights from RIAL’s application demonstrate its potential in reducing health disparities through community engaged research practices. These findings advocate for shifting from conventional techno-centric methods to participatory approaches that prioritize sustained community involvement and the cocreation of health solutions. This transition is crucial for addressing the unique challenges faced by marginalized populations and underscores the significance of contextually driven health interventions in achieving broader health equity.

## Data Availability

Data sharing is not applicable to this article as no datasets were specifically generated or analyzed during the current study. The insights and conclusions drawn are based on the synthesis of existing information and the application of the RIAL methodology, which involves qualitative assessments and processual analysis intrinsic to the project’s ongoing development. Further details on the methodology can be obtained from the corresponding author upon reasonable request.
